# Predicting major organ complications in primary Sjögren’s disease using a machine learning ensemble strategy: a dual-center retrospective clinical study

**DOI:** 10.3389/fimmu.2026.1845872

**Published:** 2026-05-29

**Authors:** Wenqi Xia, Jiayun Wu, Jingyu Zhang, Yuening Kang, Yuling Chen, Ruyi Liao, Xiaomin Li, Ya Wen, Shenghui Wen, Fanxuan Meng, Huifen Liu, Zhiyang He, Jieruo Gu, Ou Jin, Yong Ren, Qing Lv

**Affiliations:** 1 Department of Rheumatology, The Seventh Affiliated Hospital of Sun Yat-Sen University, Shenzhen, China; 2 Smart Healthcare Research Institute, Xunfei Healthcare Technology Co, Ltd, Hefei, China; 3 Department of Rheumatology, The Third Affiliated Hospital of Sun Yat-Sen University, Guangzhou, China; 4 University of Electronic Science and Technology of China, Chengdu, China; 5 Pazhou Lab, Guangzhou, China

**Keywords:** clinical study, machine learning, organ complications, primary Sjögren’s disease, prognostic indicator

## Abstract

**Objectives:**

Primary Sjögren’s disease (SjD) is a systemic autoimmune disease whose major organ complications severely affect patient prognosis. Currently, there is a lack of efficient and accurate tools for early identification of high-risk patients.

**Methods:**

This study retrospectively collected clinical data from 232 SjD patients, including demographic characteristics, clinical symptoms, and laboratory indicators. Three machine learning algorithms—logistic regression (LR), support vector machine (SVM), and random forest (RF)—were used to construct prediction models, and a soft voting ensemble strategy was applied to combine model outputs. Feature importance was analyzed using the SHAP framework.

**Results:**

The ensemble model performed best on the test set, with an AUC of 0.725, accuracy of 71%, and a negative predictive value (NPV) of 79%. SHAP analysis revealed that complement C3 and immunoglobulin G (IgG) were the most important predictors, with low C3 and high IgG levels significantly associated with complication risk.

**Conclusion:**

The ensemble model showed moderate discriminatory performance for identifying major organ complications in SjD using routinely available clinical variables. Complement C3 and IgG were identified as important predictors. The model may serve as a preliminary auxiliary risk-stratification tool, but further external validation and model optimization are required before clinical implementation.

## Introduction

Primary Sjögren’s disease (SjD) is a systemic autoimmune disease with a prevalence of 0.01–0.09% in the general population, and its incidence is gradually increasing (1). The clinical features are heterogeneous: 89–98% of SS patients present with dysfunction of exocrine glands (salivary and/or lacrimal glands), while 40–50% exhibit various major organ involvement, such as interstitial lung disease, immune-mediated cytopenia, renal tubular acidosis or renal insufficiency, and central nervous system damage (2–4). These systemic complications beyond glandular involvement often determine patient prognosis, reduce quality of life, and are major causes of mortality in SS patients (5–7). Recent studies suggest that factors such as age, disease duration, high-titer anti-La/SSB positivity, or low complement levels may be associated with major organ damage and prognosis in SS (8–10). However, early identification of patients at risk remains challenging because systemic involvement often requires comprehensive clinical, laboratory, and imaging assessments. Therefore, a simple risk-stratification model based on routinely available clinical indicators may help guide further evaluation and follow-up planning.

In recent years, artificial intelligence (AI), especially deep learning (DL), has advanced rapidly due to developments in computing and internet technologies (11). AI has shown great potential in many fields, particularly in medicine (12), where new medical models are continuously being proposed and defined. Machine learning methods are also extensively applied in rheumatology research. For example, in the study of Sjögren’s disease, to estimate the prevalence of primary Sjögren’s disease (pSS), machine learning techniques are leveraged to analyze large patient cohorts, with the aim of identifying novel biomarkers to improve diagnostic accuracy. Such research also focuses on the genetic aspects of the disease, including pathogenic mechanisms, susceptibility factors and other related topics (13). Several studies have applied machine learning approaches to classify patients with primary Sjögren’s disease (PSS) in primary care settings, aiming to facilitate early detection of the disease (14). Jordi et al. applied machine learning (ML) approaches to stratify individuals with systemic lupus erythematosus (SLE), primary Sjögren’s disease (PSS), and healthy controls. Their analysis yielded functionally coherent gene subgroups and differentially methylated CpG sites associated with enhanced predictive performance—thereby elucidating the potential pathobiological relevance of these molecular features in SLE and PSS (15). Nevertheless, no ML-based model has yet been adopted in clinical practice for early risk stratification of major organ involvement in Sjögren’s disease (SS). Although ESSDAI is widely used to assess disease activity in SjD, it requires comprehensive systemic evaluation and is not designed as a simple prediction model for early identification of major organ complications. To our knowledge, there is currently no widely accepted or externally validated clinical prediction model based on routine clinical indicators for identifying major organ complications in SjD.

In this study, we aimed to predict complications in SjD using different ML algorithms and to identify important risk factors associated with complications through optimal ML modeling. By identifying key predictors, this research may contribute to personalized management of SjD, enabling early intervention and tailored treatment and follow-up strategies to improve patient outcomes.

## Methods

**Figure 1:**
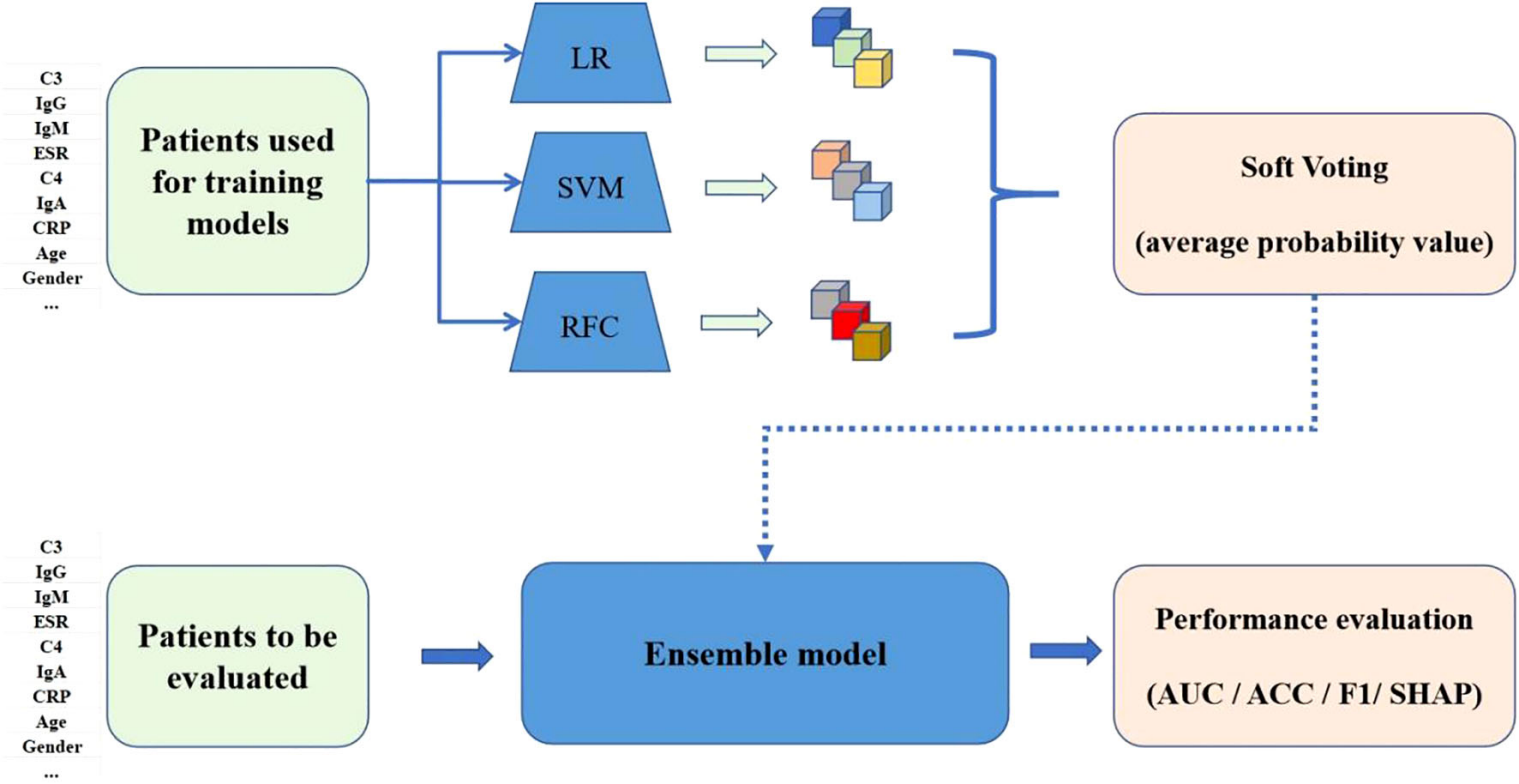
Overall flowchart.

This study included data collection, data preprocessing, dataset partitioning, model development, ensemble strategy, model evaluation, and interpretability analysis. The overall flowchart is shown in **Figure 1**.

### Data collection

This study retrospectively collected data from 232 newly diagnosed (“Newly diagnosed” was defined as patients who fulfilled the classification criteria for primary Sjögren’s disease and whose baseline clinical and laboratory data were collected at or near the time of initial diagnosis before systematic treatment) SS patients. Among them, 124 were from the Seventh Affiliated Hospital of Sun Yat-sen University (August 2020 to June 2023), and 109 were from the Third Affiliated Hospital of Sun Yat-sen University (March 2014 to March 2019). All patients met the classification established in 2022 by the American and European Consensus Group. Inclusion criteria were as follows: 1) patients fulfilling the classification criteria for primary Sjögren’s disease; 2) newly diagnosed patients evaluated at one of the two participating centers; 3) available baseline demographic, clinical symptom, and laboratory data; and 4) available systemic assessment for major organ complications. Exclusion criteria were as follows: 1) secondary Sjögren’s disease overlapping with other systemic autoimmune diseases; 2) active infection, malignancy, or other non-SjD-related causes of organ involvement; 3) prior systemic immunosuppressive treatment before baseline data collection; 4) missing key outcome information; and 5) duplicated records from the same patient.

These 232 patients were randomly divided into two sets: 160 patients in Dataset 1 for model training, and 72 patients in Dataset 2 for testing. The study was approved by the Ethics Committee of the Third Affiliated Hospital of Sun Yat-sen University (Approval No.: II2023-254-02). As a retrospective study, no patients received any reward or compensation for participation. Clinical data, systemic evaluations, and laboratory results were obtained from the hospital information system. Patients were divided into two groups based on the presence or absence of major organ complications beyond salivary and lacrimal gland involvement. Major organ complications were defined as involvement of one or more of the following systems: pulmonary, hematological, renal, and central nervous system. The definitions of major organ complications refer to the definitions of organ involvement in each system as provided in the ESSDAI, including from low activity to high activity. Pulmonary System: Encompassing interstitial pneumonia and pulmonary fibrosis diagnosed by high-resolution chest computed tomography (HRCT); Hematological System: Autoimmune thrombocytopenia, including neutropenia (neutrophil count < 1500/mm³), anemia (hemoglobin level < 120 g/L), thrombocytopenia (platelet count < 150,000/mm³), or lymphopenia (lymphocyte count < 1000/mm³); Renal System: Renal tubular acidosis, glomerular lesions (urinary protein excretion > 0.5 g/day); Central nervous system: Conditions supported by imaging studies such as neuromyelitis optica, and cerebrovascular accidents caused by vasculitis. A total of 16 feature variables were collected for all patients, including demographic characteristics (Age, Gender), clinical symptoms (Xerostomia, Keratoconjunctivitis sicca, Arthritis, Rash), and laboratory indicators (CRP, ESR, RF, ANA titer, ANA pattern, IgA, IgG, IgM, C3, C4).

### Data preprocessing

Missing values in the raw data were imputed using mean imputation. All continuous variables were standardized using Z-score normalization after imputation. Categorical variables were encoded using one-hot encoding. Continuous variables were expressed as mean ± standard deviation and compared using Welch’s t-test. Categorical variables were expressed as counts and percentages and compared using Fisher’s exact test. Variables defining major organ complications were presented descriptively and were not included in between-group statistical comparisons. A two-sided p value < 0.05 was considered statistically significant.

### Dataset partitioning and model development

The merged dataset was randomly split into a training set (70%, n=160) and a test set (30%, n=72). Three classical machine learning algorithms were used for model construction (1): Logistic Regression (LR), (2) Support Vector Machine (SVM) with a radial basis function (RBF) kernel, (3) Random Forest (RF) with 100 decision trees. All models were trained on the training set, and hyperparameters were optimized via 5-fold cross-validation. The final model performance was evaluated using a single internal test set, and no external validation cohort was available.

### Ensemble strategy

To improve predictive performance, a soft voting-based ensemble strategy was employed. The ensemble model took the predicted probabilities from the three base models (LR, SVM, RF) on the test set as input and computed a weighted average probability (with equal weights assigned to each model in this study) to produce the final ensemble prediction.

### Model evaluation

The area under the receiver operating characteristic curve (AUC) was the primary metric for evaluating model performance. Additional metrics included accuracy (ACC), precision (PPV), recall (sensitivity, SENS), specificity (SPEC), F1-score, and negative predictive value (NPV). All evaluations were performed on the test set.

### Interpretability analysis

To understand the model decision mechanism and identify key predictors, the SHAP (Shapley Additive Explanations) framework was applied. Based on game theory, SHAP quantifies the contribution of each feature to the prediction outcome for individual samples, providing both global and local interpretability of the model output.

## Results

### Baseline characteristics of the study population

Demographically, the male-to-female ratios in the training and testing sets were consistent with disease characteristics (training set: 3/60 and 6/91; testing set: 2/29 and 5/36). The mean ages across subgroups were similar (44–49 years). Regarding organ involvement, hematological system involvement was most prominent (85.7% in training, 48.4% in testing), followed by pulmonary (14.3% training, 35.5% testing) and renal (9.5% training, 29.0% testing) involvement. Differences in organ involvement patterns were observed between the training and test sets. Hematological involvement was more frequent in the training set, whereas pulmonary and renal involvement accounted for a higher proportion of complications in the test set. These differences suggest heterogeneity in the distribution of organ involvement subtypes between the two datasets.

In the training set, patients with major organ complications had significantly higher IgG levels and ESR, and rash was more frequent compared with patients without complications. In the testing set, patients with major organ complications had significantly higher IgA levels and lower C3 levels, whereas arthritis was less frequent in the complication group. Variables defining major organ complications were presented descriptively and were not subjected to statistical comparison because they formed part of the outcome definition. Baseline characteristics are summarized in **Table 1**.

**Table 1:** Baseline characteristics of patients.

Characteristic​	Training group (n=160)		P value	Testing group(n=72)		P value
	With Complications (n=63)	Without Complications (n=97)		With Complications (n=31)	Without Complications (n=41)	
Gender (Male/Female)​	3/60	6/91	1.000	2/29	5/36	0.691
Age (years)​	44.94 ± 10.44	45.66 ± 11.64	0.684	47.55 ± 16.32	48.95 ± 12.81	0.695
Organ involvement						
Pulmonary	9/63	0/97	/	11/31	0/41	/
Hematological System	54/63	0/97	/	15/31	0/41	/
Renal	6/63	0/97	/	9/31	0/41	/
Nervous System	1/63	0/97	/	2/31	0/41	/
Clinical symptoms						
Xerostomia	33/63	47/97	0.746	21/31	27/41	1.000
Keratoconjunctiv-itis Sicca	20/63	41/97	0.188	16/31	25/41	0.477
Arthritis​​	26/63	44/97	0.629	14/31	31/41	**0.013**
Rash​​	17/63	9/97	**0.004**	6/31	4/41	0.310
Immunological and inflammatory indicators						
- IgA (g/L)	3.51 ± 2.81	2.94 ± 1.17	0.131	2.96 ± 1.35	2.40 ± 0.85	**0.048**
- IgG (g/L)	20.58 ± 8.27	16.77 ± 6.47	**0.003**	20.57 ± 22.28	15.52 ± 5.05	0.225
- IgM (g/L)	1.50 ± 1.18	1.25 ± 0.88	0.152	1.27 ± 1.08	1.19 ± 0.62	0.714
- C3 (g/L)	0.98 ± 0.24	1.03 ± 0.19	0.166	0.91 ± 0.24	1.07 ± 0.22	**0.005**
- C4 (g/L)	0.22 ± 0.09	0.23 ± 0.08	0.475	0.21 ± 0.08	0.24 ± 0.10	0.162
- CRP (mg/L)	8.57 ± 24.25	3.60 ± 8.31	0.121	2.92 ± 3.92	5.78 ± 10.71	0.121
- ESR (mm/h)	48.08 ± 36.41	31.63 ± 26.60	**0.003**	40.37 ± 30.60	29.45 ± 22.44	0.100
- RF (IU/ml)	94.73 ± 225.96	82.34 ± 269.40	0.754	62.05 ± 92.13	142.9 ± 380.57	0.197

Continuous variables are presented as mean ± standard deviation and were compared using Welch’s t-test. Categorical variables were compared using Fisher’s exact test. Organ involvement variables were not statistically compared because they are components of the outcome definition for major organ complications. Bold values indicate p < 0.05.

### Predictive performance of machine learning models

**Figure 2:**
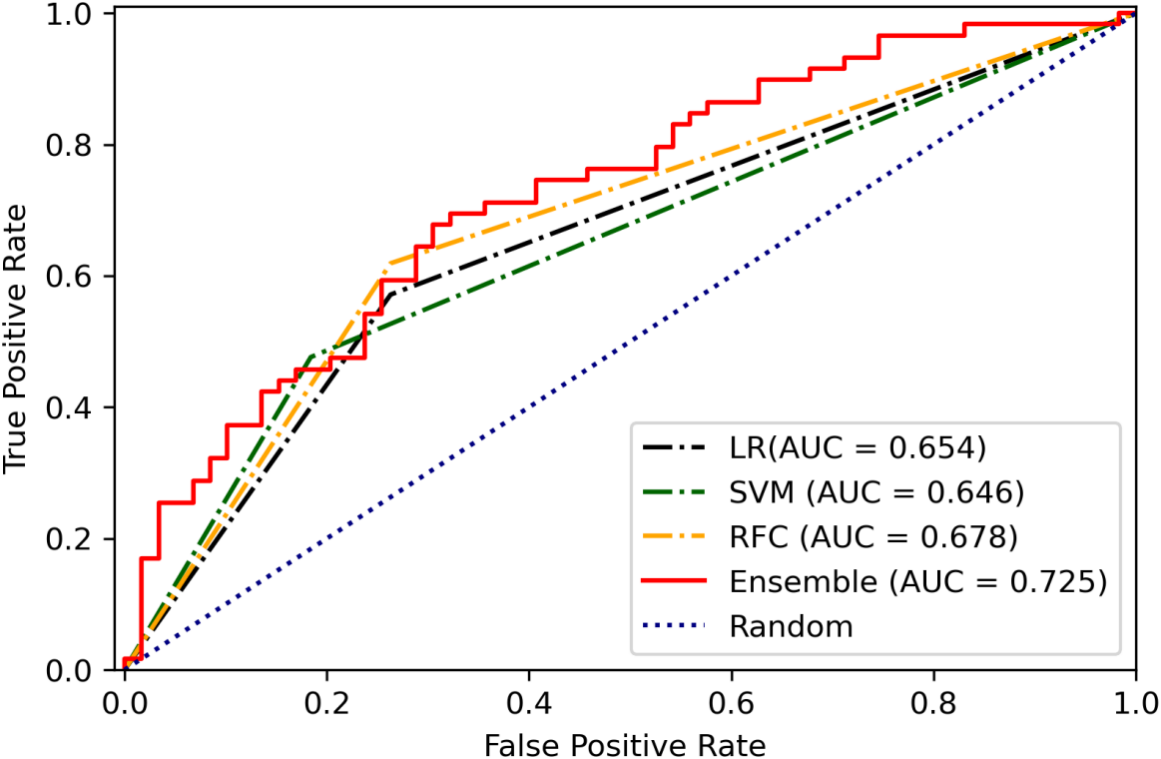
ROC curves and AUC values of different prediction models on the test- set.

To evaluate the effectiveness of different machine learning algorithms in predicting complication risk in SjD, we constructed LR, SVM, and RF models. Their performance on the independent test set is shown in **Figure 2** and **Table 2**.

**Table 2:** Performance metrics of different prediction models on the test set.

Models	AUC	ACC	F1	PPV	NPV	SENS	SPEC
Logistic Regression (LR)​	0.654	0.66	0.66	0.55	0.76	0.55	0.74
Support Vector Machine (SVM)​	0.646	0.67	0.67	0.58	0.74	0.48	0.82
Random Forest Classifier (RFC)​	0.678	0.69	0.69	0.57	0.78	0.62	0.74
Ensemble Strategy	0.725	0.71	0.71	0.59	0.79	0.56	0.79

The LR model achieved an AUC of 0.654. The SVM model performed slightly worse, with an AUC of 0.646. The RF model showed the best discriminative ability among the three, with an AUC of 0.678.

To further improve performance, an ensemble strategy was used to combine the predicted probabilities of the three base models. The ensemble model achieved the best predictive performance, with a significantly improved AUC of 0.725. Other performance metrics were as follows: accuracy (ACC) 0.71, F1-score 0.71, positive predictive value (PPV) 0.59, negative predictive value (NPV) 0.79, sensitivity (SENS) 0.56, and specificity (SPEC) 0.79. The ensemble model achieved the highest AUC among the evaluated models, with an AUC of 0.725. However, this result indicates moderate discrimination and should be interpreted cautiously given the limited sample size and single internal train-test split. The relatively high NPV and specificity suggest potential value for preliminary low-risk identification, whereas the modest sensitivity indicates that the model should not be used as a stand-alone tool to exclude major organ complications.

### Interpretability analysis of the model

**Figure 3:**
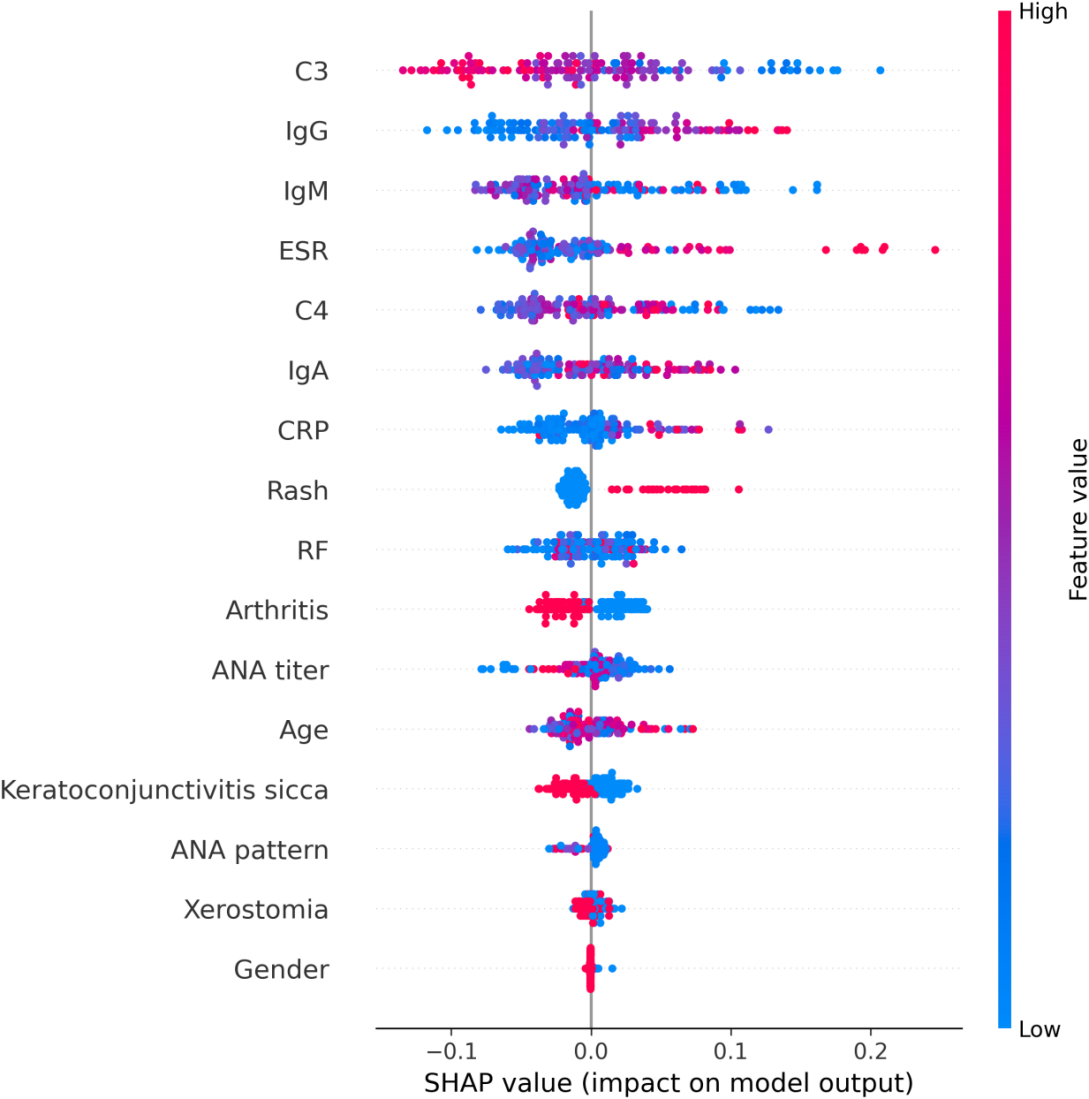
Feature importance ranking based on SHAP values.

To interpret the decision mechanism of the best-performing model (ensemble model) and identify key predictors, we performed interpretability analysis using the SHAP framework. The feature importance ranking based on SHAP values is shown in **Figure 3**.

The analysis revealed that complement C3 and immunoglobulin G (IgG) were the two most important predictive features. SHAP dependence plots showed that lower C3 levels were significantly associated with higher complication risk (positive SHAP values). Similarly, higher IgG levels contributed positively to the model output, indicating that they are important risk factors for complications. Other features such as age, immunoglobulin A (IgA), and C-reactive protein (CRP) also played significant roles in the prediction.

SHAP analysis provided an interpretable ranking of variables contributing to model output. Lower C3 and higher IgG levels were associated with increased predicted risk of major organ complications, supporting the biological plausibility of the model findings.

## Discussion

This study successfully constructed a machine learning model to predict the risk of complications in primary Sjögren’s disease (SjD) by integrating multicentric clinical data and employing an ensemble learning strategy. Compared with the established Sjögren’s disease activity index (ESSDAI), the proposed model facilitates earlier identification of patients at risk for systemic organ involvement. The research yielded several innovative results:

First, key findings and model performance interpretation: The ensemble model significantly improved predictive performance. Among single models, random forest (RFC, AUC = 0.678) performed best, while the ensemble model combining LR, SVM, and RFC improved AUC to 0.725 (a 7.0% increase). This improvement stems from the ensemble strategy, which effectively integrates the discriminative characteristics of different algorithms through weighted average probability: LR captures linear feature-outcome relationships, SVM handles nonlinear boundaries via kernel functions, and RFC identifies high-dimensional feature interactions. This “diversity complementarity” mechanism reduces the bias-variance trade-off, resulting in stronger generalization ability on the independent test set. Clinical utility validation: The high negative predictive value (NPV = 0.79) and specificity (SPEC = 0.79) of the ensemble model indicate its reliability in excluding low-risk patients. Although the positive predictive value (PPV = 0.57) suggests that final judgment should combine clinical assessment, its sensitivity (SENS = 0.62) is significantly higher than traditional risk assessment methods. This supports the model’s potential as an effective auxiliary tool in clinical decision-making. Ensemble learning strategies are widely recognized as a robust machine learning methodology, distinguished by their high predictive accuracy, capacity to handle complex interactions, and resistance to overfitting. Their better performance has been consistently demonstrated across various medical domains. In the development of predictive models for colon cancer, ensemble models have proven more effective in distinguishing between high-risk and low-risk patients compared to individual staging or recurrence prediction models (16). Mondal SS integrated two deep learning models for diabetic retinopathy detection and found that the ensemble approach achieved superior predictive performance compared to any single constituent model (17). For early diagnosis of brain tumors, a weighted-average ensemble deep learning model was proposed for tumor classification. By leveraging the strengths and compensating for the weaknesses of multiple models, the ensemble framework enhanced classification accuracy (18). These collective findings not only underscore the versatility and reliability of ensemble models in managing heterogeneous clinical datasets but also reinforce their practical utility in addressing complex predictive tasks across various medical specialties. In view of the superior performance of the integrated model, we employed a machine learning approach to develop the model even with a relatively small sample size in the present research. Additionally, it has been widely documented that machine learning methods can achieve valid analytical outcomes in research with limited sample sizes. Noguchi, Keigo et al. obtained tongue photographs of 60 patients to clarify the relationship between the features of the tongue and SS (19). A prospective study enrolling 80 participants was performed to reveal an immuno-lipidomic signature in labial salivary gland that accurately distinguishes early primary Sjögren’s syndrome from other causes of sicca symptoms by using machine learning (20). The novelty of our study lies in developing an interpretable model based on routinely available clinical and laboratory variables for major organ complication risk stratification in SjD.

Second, the results of this study may offer new insights for the treatment and management of SS patients. The model can effectively identify patients unlikely to develop complications, thereby avoiding unnecessary medical resource use and reducing repeated systemic screenings (especially radiation-based exams like chest CT) for low-risk patients. It also provides a new reference for prognosis assessment and follow-up strategy: high-risk patients may require more aggressive treatment and closer monitoring, while low-risk patients can have extended follow-up intervals, avoiding excessive treatment and drug side effects. Furthermore, it offers specialized assistance to non-specialist or primary care physicians, prompting closer complication screening for high-risk patients and reducing delays due to lack of specialist experience.

Third, this study used an AI model to analyze correlations between complications and high-risk factors in SS patients, revealing that certain clinical features are closely associated with complication occurrence. C3 and IgG were important model contributors and may reflect systemic immune activation involving B-cell hyperactivity and complement consumption (21). C3, a central component of complement activation, decreases (positive SHAP value) reflect ongoing consumption via classical or alternative pathways, suggesting that abnormal complement activation may be a key driver of multi-system damage (22, 23). Studies have indicated that persistent serological activity, such as low complement C3 and hypergammaglobulinemia, can increase the ESSDAI score in Sjögren’s disease (24), while patients with ILD are also associated with low C3 levels (25). These findings are consistent with the conclusions of our study. The dual role of elevated IgG: high IgG levels (positive SHAP value) are not only a marker of B-cell overactivation but also directly contribute to vasculitis and neuropathy via immune complex deposition (26–28). Notably, IgG had a higher predictive weight than IgA or IgM in this study, supporting the hypothesis of IgG subtype specificity in SjD organ damage (29).

Fourth, the AI model developed in this study for predicting major organ complications in SS patients has potential for broad application. The clinical data used are routinely collected during SS follow-up, such as symptoms, immunoglobulins, and complement levels. These data are easy to obtain, low-cost, and accessible even in primary care and underdeveloped regions, facilitating model promotion and application.

Despite these innovative results, this study has limitations that should be addressed in future research. The sample size of this study was relatively limited compared with the number of candidate predictors and machine learning models evaluated. Although the included models were classical machine learning algorithms rather than highly parameterized deep learning models, the use of multiple models and an ensemble strategy in a dataset of 232 patients may still increase the risk of overfitting and model instability. In addition, the dataset was split once into a 70% training set and a 30% test set. Although 5-fold cross-validation was used for hyperparameter optimization within the training set, repeated cross-validation, bootstrap internal validation, and external validation were not performed. Therefore, the reported model performance may be affected by the specific random split and should be interpreted cautiously. Future studies should validate and update the model in larger prospective multicenter cohorts.

Secondly, the imbalance in organ involvement patterns between the training and test sets may have influenced model performance and generalizability. SjD-related major organ complications are clinically heterogeneous, and hematological, pulmonary, renal, and neurological involvement may have distinct biological characteristics and predictors. Therefore, the performance of a single model trained on mixed organ involvement phenotypes may vary across different patient subsets. Future studies with larger samples should consider organ-specific modeling or stratified validation. Additionally, the model was only tested on one internal validation set; its reliability and validity require further verification through multicenter, large-sample clinical studies. Another limitation is the potential heterogeneity between the two centers. Patients were collected from different hospitals and different time periods, which may have introduced differences in disease spectrum, referral patterns, examination strategies, and laboratory measurement practices. Because center-specific validation was not performed, the influence of center-related heterogeneity on model performance cannot be fully excluded. Future studies should include larger external cohorts and perform center-stratified or leave-one-center-out validation to further assess model generalizability.

The handling of missing data is another important limitation, the proportion of missing data observed in this study was minimal; therefore, in this study, missing continuous variables were imputed using mean imputation. Although this method is simple and commonly used in retrospective machine learning studies, it may reduce data variability and introduce potential bias, particularly when missingness is not completely random. Because additional model re-training using alternative imputation strategies was not performed, the potential influence of missing-data handling on model performance cannot be fully excluded. Future studies with larger prospective cohorts should use more robust imputation methods, such as multiple imputation, and compare model performance across different missing-data handling strategies.

Furthermore, the sensitivity of the ensemble model was relatively low, indicating that some patients with major organ complications may be misclassified as low risk. Therefore, the model should not replace comprehensive clinical evaluation, the model is better positioned as an auxiliary tool for preliminary risk stratification, The PPV and NPV also should be interpreted in the context of disease prevalence and class distribution. In this cohort, the NPV of 0.79 suggests that the model may be more useful for supporting preliminary low-risk identification, whereas the modest PPV indicates that positive predictions require further clinical confirmation. More importantly, the relatively low sensitivity limits the model, s ability to detect all high-risk patients. Therefore, model predictions should be combined with clinical symptoms, physician assessment, and targeted examinations.

Finally, Autoantibodies are central to the immunopathogenesis and classification of SjD. Anti-SSA/Ro and anti-SSB/La antibodies are closely related to B-cell activation and may be associated with systemic manifestations in a subset of patients. Previous studies have revealed that individuals with active immune features tend to develop a wide range of systemic complications (30). Therefore, the finding that high IgG and low C3 contributed to model prediction should not be interpreted as completely independent of autoantibody-mediated immune activation. Instead, these variables may reflect a broader immunological axis involving autoantibody production, hypergammaglobulinemia, immune-complex formation, and complement consumption. In this context, IgG and C3 may provide clinically accessible downstream indicators of systemic immune activation. However, because anti-SSA/Ro and anti-SSB/La antibody titers were not incorporated into the machine learning model, the incremental predictive value of IgG and C3 beyond autoantibody status could not be fully assessed. Future studies should integrate autoantibody profiles, antibody titers, complement levels, and immunoglobulin levels into a multifactorial model to better characterize the immunological risk pattern of major organ complications in SjD.

## Conclusion

This study developed an interpretable machine learning model for preliminary identification of major organ complications in patients with SjD using routinely available clinical variables. The ensemble model achieved the highest AUC among the evaluated models in the internal test set, but its overall performance remained moderate and its sensitivity was limited. Complement C3 and IgG were important contributors to the model and may reflect systemic immune activation in SjD. However, their predictive value should be interpreted in the context of autoantibody-mediated immune responses, and future models should incorporate anti-SSA/Ro and anti-SSB/La profiles. Given the limited sample size, single internal validation, and lack of external validation, the model should be regarded as an exploratory risk-stratification tool. Larger prospective multicenter studies are needed to validate and optimize the model before clinical application. Future research will focus on optimizing the study design, expanding the sample size, supplementing more predictive variables, and establishing a multifactorial prediction model to guarantee good general applicability.

## Data Availability

The original contributions presented in the study are included in the article/supplementary material. Further inquiries can be directed to the corresponding authors.
